# Evaluation of the functional efficacy of an antioxidative probiotic in healthy volunteers

**DOI:** 10.1186/1475-2891-4-22

**Published:** 2005-08-04

**Authors:** Epp Songisepp, Jaak Kals, Tiiu Kullisaar, Reet Mändar, Pirje Hütt, Mihkel Zilmer, Marika Mikelsaar

**Affiliations:** 1 Department of Microbiology, University of Tartu, 50411 Tartu, Estonia; 2 Department of Biochemistry, University of Tartu, 50411 Tartu, Estonia

## Abstract

**Background:**

In persons without clinical symptom it is difficult to assess an impact of probiotics regarding its effect on health. We evaluated the functional efficacy of the probiotic *Lactobacillus fermentum *ME-3 in healthy volunteers by measuring the influence of two different formulations on intestinal lactoflora, fecal recovery of the probiotic strain and oxidative stress markers of blood and urine after 3 weeks consumption.

**Methods:**

Two 3-week healthy volunteer trials were performed. Open placebo controlled (OPC) study participants (n = 21) consumed either goat milk or by *L. fermentum *ME-3 fermented goat milk (daily dose 11.8 log CFU (Colony Forming Units). Double blind randomised placebo controlled (DBRP) study participants (n = 24) received either capsules with *L. fermentum *ME-3 (daily of dose 9.2 CFU) or placebo capsules.

The faecal lactoflora composition, faecal ME-3 recovery, effect of the consumption on intestinal lactoflora, and oxidative stress markers of blood (total antioxidative activity; total antioxidative status and glutathione red-ox ratio) was measured.

**Results:**

ME-3 was well tolerated and a significant increase in total faecal lactobacilli yet no predominance of ME-3 was detected in all study groups. Faecal recovery of ME-3 was documented by molecular methods only in fermented milk group, however the significant improvement of blood TAA (Total Antioxidative Activity) and TAS (Total Antioxidative Status) indices was seen both in case of fermented goat milk and capsules", yet glutathione re-ox ratio values decreased only in case of fermented by ME-3 goat milk.

**Conclusion:**

The functional efficacy of both consumed formulations of an antioxidative probiotic *L. fermentum *ME-3 is proved by the increase of the intestinal lactobacilli counts providing putative defence against enteric infections and by reduction of the oxidative stress indices of blood and urine of healthy volunteers. In non-diseased host the probiotic health claims can be assessed by improvement of some measurable laboratory indices of well-established physiological functions of host, e.g. markers of antioxidative defence system.

## Background

Probiotics are defined as live microbial food supplements, which beneficially influence human health [1,2]. Widely accepted probiotics contain different lactic acid producing bacteria of human origin: bifidobacteria, lactobacilli or enterococci. Nowadays the concept of functional foods, incl. probiotic food and dietary supplements implies to their ability to beneficially influence body functions in order to improve the state of well-being and health and reduce the risk of disease [2,3]. The important areas of human physiology that are relevant to functional food science according ILSI (International Life Science Institute) and FUFOSE (The European Commission Concerted Action on Functional Food Science in Europe) are besides others, the modulation of basic metabolic processes and defence against high-grade oxidative stress [4,5].

Human nutrition is clearly associated with oxidative metabolism, which beside production of energy is involved in a number of vital functions of the host. For example, under physiological conditions the reactive species (including peroxyl radicals, nitric oxide radical, superoxide anion) figure a crucial role in primary immune defense of the human body by phagocytic cells against harmful microorganisms [6,7]. On the other hand, a prolonged excess of reactive species is highly damaging for the host biomolecules and cells, resulting in dysbalance of the functional antioxidative network of the organism and leading to substantial escalation of pathological inflammation [8].

By our knowledge, no systematic studies have been performed to approve the functional efficacy of different formulations of probiotic on the antioxidative defence system of a healthy human. In our previous study *Lactobacillus fermentum *ME-3 (DSM 14241) [9-11], expressed strong antimicrobial activity against Gram-positive and Gram-negative entero- and uropathogens [12,13]. The cells and cell lysate of *L. fermentum *ME-3 possessed substantial antioxidative potency [14]. In an animal experiment ME-3 suppressed the excessive oxidative stress reaction caused by *Salmonella *infection in intestinal mucosa and thus improved the gut mucosal antioxidative status [15]. The antioxidative effect of *L. fermentum *ME-3 on human body oxidative stress markers was confirmed by our pilot study with fermented goat milk [16].

The aim present study was to evaluate the functional efficacy of the probiotic strain *L fermentum *ME-3 in the human gastrointestinal tract (GIT) of healthy volunteers. The faecal recovery, effect of two different formulations on total faecal lactoflora and oxidative stress markers of blood and urine were compared after 3 weeks consumption.

## Methods

### Formulations

The efficacy of two different formulations (experimental fermented goat milk and probiotic capsules) on the human body oxidative stress markers was evaluated. *Lactobacillus fermentum *ME-3, a probiotic strain of healthy human intestinal origin [17], has been identified by biochemical and molecular methods [9]. The patent application has been submitted to the Estonian Patent Agency (Application No. 0356/01PV) as well as to the International Bureau of World Intellectual Property

Organization (WIPO) (Application No. WO03002131) [11]. *L. fermentum *ME-3 was used as freeze-dried powder in capsulated form and in fermented milk.

#### Capsules

Gelatine coated capsules were manufactured by the Tallinn Pharmaceutical Company. The freshly prepared probiotic capsules contained 9.0 logs CFU of *L. fermentum *ME-3 per capsule in addition to 250 mg of saccharose and microcellulose. Identical placebo capsules contained only saccharose and microcellulose. All capsules were stored at +4°C.

#### Survival of ME-3 in capsule

Survival of ME-3 in capsule was monitored during 12 months at +4°C. The content of one capsule was dissolved aseptically in 2 ml of 0.9% NaCl solution. The suspension was vortexed, serially diluted and seeded 0.1 ml on de Man-Rogosa-Sharpe (MRS) agar medium (OXOID, U.K.) and incubated 48 hours at 37°C microaerobically (10% CO_2_). The number of colonies was counted and the viable cell count in capsule was calculated.

#### Experimental fermented milk

Three different lots of experimental fermented goat milk was prepared for the 3-week trial with healthy volunteers in order to establish the health effects of ME-3 consumption. The study group was supplied with fresh product once a week. Experimental fermented milk was prepared as described previously [16] by combining the probiotic strain with two supportive lactobacilli cultures *L. plantarum *LB-4 and *L. buchneri *S-15. *L. buchneri *strain S1-5 decreased the specific taste of the goat milk. *L. plantarum *LB-4 was included as a strong producer of exopolysaccharides, which gives the fermented milk a cream-like consistence and delightful acidity. The goat milk was inoculated with 2% mixture of *Lactobacillus *strains and incubated at 37°C for 24 hours. Theproduct, ready to use, was cooled and stored at 4°C.

#### Survival of L. fermentum ME-3 in fermented goat milk

To measure the viable cell count of ME-3 in fermented goat milk, samples were taken at the end of fermentation (before cooling the product), and after 24 h, 32 h, 48 h and 7 days from the preparation, when the product was stored at 4°C. The amount of 0.5 ml of the fermented milk was serially diluted in saline and plated on MRS agar medium and incubated for 48 h at 37°C in microaerobic conditions.

### Design of human volunteer trials

Two healthy volunteer (n 45) trials, particularly open placebo controlled (OPC) study and double blind randomized placebo controlled (DBRP) study were carried on to evaluate the functional efficacy of *L. fermentum *ME-3 in the human body. The inclusion criteria included the wish to participate, no known health problems, and no medical conditions requiring drug therapy, no other yogurts or no special diets. The subjects with a history of GIT disease, food allergy and acute infection, use of any antimicrobial agent within the last month or use of any regular concomitant medication including antioxidant vitamins and anti-inflammatory non-steroidal drugs were excluded.

The members of the trial were daily questioned about their general welfare, intestinal function (general welfare, gut gas production, stool frequency) and putative adverse effects. The withdrawal criteria from the trials included acute infections during the study. Reasons for dropout were the unwillingness to proceed with the study or relocation to new area. The blood samples (6 ml) from the antecubital vein, faecal and urine samples were collected before and at the end of all clinical trials. Participants of all trials gave informed consent to the study protocols approved by the Ethical Committee of Tartu University.

#### Open placebo controlled fermented goat milk trial

The study participants were 5 men and 16 women, mean age 50 years (range 35–60). During three weeks of the trial the study group (3 males and 13 females) consumed daily 150 ml fermented goat milk. The daily dose of probiotic *Lactobacillus *strain was 11.2 to 11.8 log CFU per person. The control group (1 male and 4 females) consumed the same dose of fresh goat milk.

#### Probiotic capsule trial

A DBRP study (ISRCTN 53164826) was carried out as follows. The study group consisted of 15 men and 9 women, mean age 52 years (range 40–60) allocated according to their wish to participate and randomly divided by an independent person and computer program for two groups. The study group members (8 males and 4 females) took three probiotic containing capsules (8.4 log CFU per capsule) two times daily (the daily dose 9.2 log CFU) during three weeks. The placebo group (7 males and 5 females) received identical capsules without the probiotic strain.

Faecal samples of all participants to assess change in faecal lactoflora and the persistence of the ingested probiotic strain were collected before and at the end of trial. Several laboratory indices of blood and urine were measured before and after the consumption of ME-3. Here we report on changes in human body oxidative stress markers as total antioxidative activity (TAA), total antioxidative status (TAS) and glutathione red-ox ratio (GSH/GSSG) from blood serum.

### Microbiological analyses of faeces

The total count of lactobacilli and the count of *L. fermentum *were evaluated in faecal samples. The faecal samples were collected at day 0 and 21 in both trials. Samples were kept at -80°C before analyzed. Serial dilutions (10^-2^–10^-9^) of the weighed faecal samples were prepared with phosphate buffer (pH 7.2) and 0.05 ml of aliquots was seeded onto MRS agar medium [17]. The plates were incubated at 37°C for 4 days microaerobically in 10% CO_2 _environment (incubator IG 150, Jouan, France). The catalase negative colonies were selected on the basis of typical for LAB colony morphology, cells microscopy and Gram staining.

The count of *Lactobacillus *species was expressed in log_10 _colony forming units per gram faeces (log_10 _CFU/g) and percentage (relative share) in the total count of lactobacilli. The detection level of lactobacilli was a 3.0 log CFU/g faeces.

The relative amount of *L. fermentum*, colonizing the gastrointestinal tract of persons in the study groups was expressed as a proportion of the total count (%), using the Bioquant program [18]). The program gives output data for every microorganism as an absolute count (log_10 _CFU/g) and their percentage in the total count with its normal values.

### AP-PCR typing

The putative ME-3 isolates were typed by arbitrarily primed polymerase chain reaction (AP-PCR). Genomic DNA was extracted from 24 h old cultures, cultivated on MRS agar microaerobically with the QIAamp DNA Mini Kit 50 (QIAGEN GmbH., Hilden, Germany) according to the manufacturers instructions. AP-PCR typing was done with two primers: ERIC1R (5'-ATGTAAGCTCCT GGGGATTCAC-3') and ERIC2 (5'-AAGTAAGTGACTGGGGTGAGCG -3') (DNA Technology A/S, Aarhus, Denmark). A 30 μl volume of reaction mixture consisted of 10 × PCR buffer (Fermentas, Vilnius, Lithuania), 2.5 mM MgCl_2 _(Fermentas, Vilnius, Lithuania), 200 μM deoxynucleoside triphosphate mixture (dATP, dGTP, dTTP and dCTP, Amersham Pharmacia Biotech, Freiburg, Germany) 0, 60 μg of each primer and 2.5 U Taq DNA Polymerase (Fermentas, Vilnius, Lithuania,) and 5 μl of extracted DNA according to Matsumiya *et al*. [19]. The PCR mixture was subjected to thermal cycling 35 cycles of denaturation at 95°C for 1 min, annealing at 35°C for 1 min, and extension at 74°C for 2 min, with a final extension at 74°C for 5 min with the PTC-200 thermal cycler (Eppendorf AG, Hamburg, Germany). The PCR products were separated by electrophoresis in a horizontal 2% agarose gel containing 0.1 μl/ml ethidium bromide in Tris-acetic acid-EDTA (TAE) buffer (40 mM Tris, 20 mM boric acid, 1 mM EDTA, pH 8.3) (Bio-Rad Laboratories, Hercules, USA) at constant voltage of 120 V. A 1 kb ladder (GeneRuler, Fermentas, Vilnius, Lithuania) was used as a base pair size marker. The banding patterns of isolates were visualized with UV light and compared with that of *L. fermentum *ME-3 strain.

### Measurement of human body oxidative stress status

Blood serum was analysed for total antioxidative activity TAA, total antioxidative status TAS and glutathione red/ox ratio (oxidized glutathione and reduced glutathione, GSSG/GSH). TAA of the serum was assessed by the linolenic acid test (LA-test) described previously [16]. This test evaluates the ability of the sample to inhibit lipid peroxidation. TAS of the serum was measured with a commercially available kit (TAS, Randox Laboratories Ltd. Ardmore, UK) as described elsewhere [16], water-soluble vitamin E (Trolox) serving as a standard. This method is based on the inhibition of the absorbance of the ferrylmyoglobin radicals of 2,2'-azinobis-ethylbenzothiazoline 6-sulfonate (ABTS+) generated by activation of metmyoglobin peroxidase with H_2_O_2_.

The cellular oxidative stress markers as total glutathione and oxidized glutathione were measured using the method of Griffith [20] as described elsewhere [16]. The glutathione content was calculated on the basis of a standard curve generated with known concentration of glutathione. Amount of GSH (μg/ml) was calculated as a difference between the total glutathione and GSSG (total glutathione – GSSG). The glutathione red/ox ratio was expressed as GSSG/GSH.

### Statistical Analysis

The computer program Sigma Stat for Windows 2.0 (Jandel Corporation, USA) was applied. The counts of faecal lactoflora were compared by using Student's t-test and Mann-Whitney rank sum test. Changes in oxidative stress markers of blood sera (TAA, TAS and glutathione red-ox ratio) were evaluated by Student's t-test, paired t-test and Mann-Whitney rank sum test. The choice of tests was made automatically according to the distribution of the data. Both microbial and biochemical markers were given as mean and standard deviation.

One-way ANOVA test was performed to compare the effect of different formulation on TAA, TAS and faecal lactoflora parameters.

Differences were considered statistically significant if the value was p < 0.05.

## Results

### Survival of ME-3 in formulations

In capsule after approximately 1-log drop after one week from the production of the capsules, the viable count of the probiotic strain remained stable at the level of 8.4 log CFU per capsule. Additional results have shown at +4°C the stability of the freeze-dried capsulated culture at least 17 months from the production.

In fermented goat-milk the cell count of the probiotic strain varied insignificantly from 9.0 to 9.7 log CFU/ml from one preparation to the other. The viable count of ME-3 in the fermented goat milk was found to remain stable at least during 7 days of storage at 4°C.

### Human volunteer trials

No dropouts were registered during volunteer trials, yet one participant was withdrawn from the probiotic capsule trial due to acute respiratory viral infection. Besides, no adverse affects in general welfare or changes in GI functionality were assessed during the trial.

#### Changes in total LAB count

**Figure 1:**
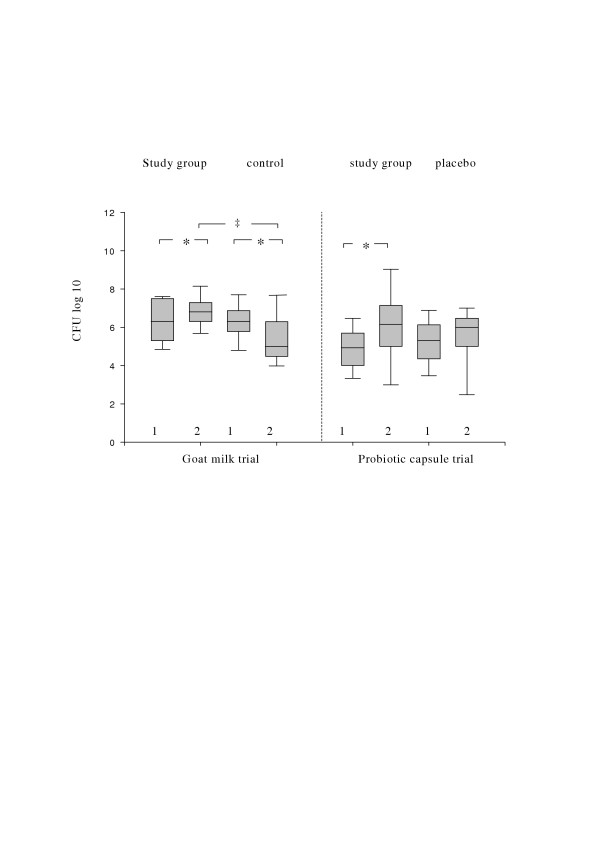
**Increase of total fecal counts of lactobacilli in healthy volunteers consuming of ME-3 in fermented goat milk and probiotic capsule. **1 – Day 0, 2 – Day 21 Significantly different from pre-treatment values (Student's t-test): * p < 0.05; Significantly different from control (Student's t-test): ‡ p = 0.01

The consumption of both ME-3 fermented milk and ME-3 capsule significantly increased the total count of lactobacilli in faeces as compared to the initial levels (Fig. 1). In opposite, in the group of volunteers consuming non-fermented goat-milk there was even a decrease in total LAB counts during the 3-week trial and no changes were found in capsule placebo group.

#### Recovery of the probiotic strain

**Figure 2:**
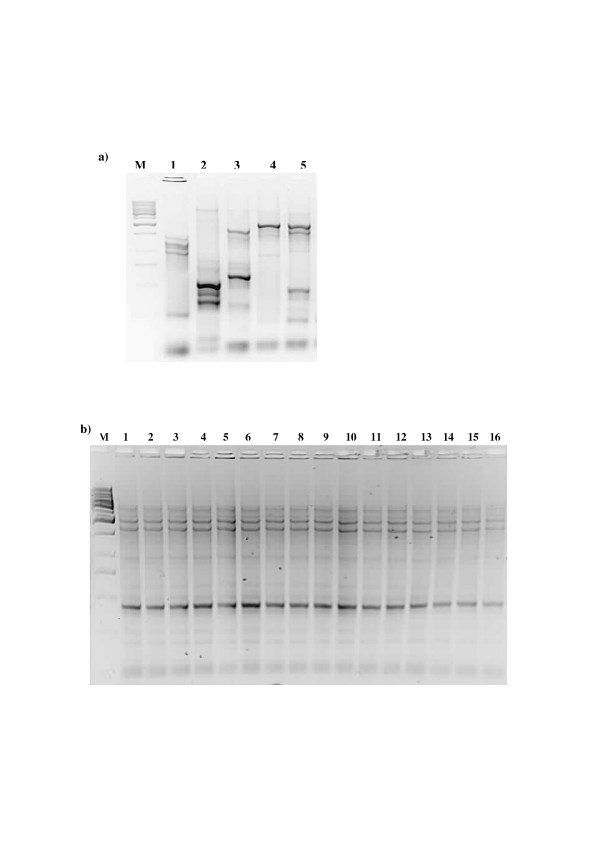
**Confirmation for the survival of *L. fermentum *ME-3 in GIT in subjects receiving ME-3 fermented goat milk by AP-PCR in a horizontal 2% agarose gel. **a) From the left: M – DNA 1 kb Ladder, Line 1 -*L. bervis *ATCC 14869, Line 2 – *L. buchneri *ATCC 4005, Line 3 – *L. reuteri *DSM 20016, Line 4 – *L. fermentum *ATCC 14931, Line 5 – *L. fermentum *ME-3 b) From the left: M – molecular weight marker, Line 1 ...16 – ME-3 like profiles from feces of goat milk trial study group participants

In goat milk group *L. fermentum *as a species appeared in fecal samples of all individuals (n = 16) after consumption of fermented goat milk (Table 1). The AP-PCR confirmed the recovery of ME-3 in the faeces of all study group members (Fig. 2). However, in different trials the administration of ME-3 strain did not lead to the predominance of *Lactobacillus fermentum *species (Table 1).In the probiotic capsule trial the strain ME-3 was not detectable between *L. fermentum *isolates by AP-PCR.

**Table 1:** Changes in fecal recovery of *L. fermentum *during healthy human volunteer trials

	*L. fermentum*					
Groups	* Prevalence (%)		† Count (log10)		^‡^Proportion (%)	
	Day 0	Day 21	Day 0	Day 21	Day 0	Day 21
Goat milk trial, ME-3 (n = 16)	25 (4/16)	100 (16/16)	7.0 ± 0.7	7.3 ± 1.4**	21	13
Control (n = 5)	-	20 (1/5)	-	3.6	-	28
Capsule trial, ME-3 (n = 11)	16.7(2/12)	33.3 (2/12)	4.3 ± 0.5	5.8 ± 1.6	4	9
Placebo (n = 12)	25 (3/12)	16.7 (2/12)	6.3 ± 2.5	8.0 ± 1.6	11	19

* Percentage of subjects with fecal *L. fermentum *inside the group

** Significantly different from the pre-treatment values (paired t-test): p < 0.001

^† ^Median value ± SD

^‡ ^Proportion of *L. fermentum *among fecal LAB

#### Antioxidative health effect of ME-3

**Figure 3:**
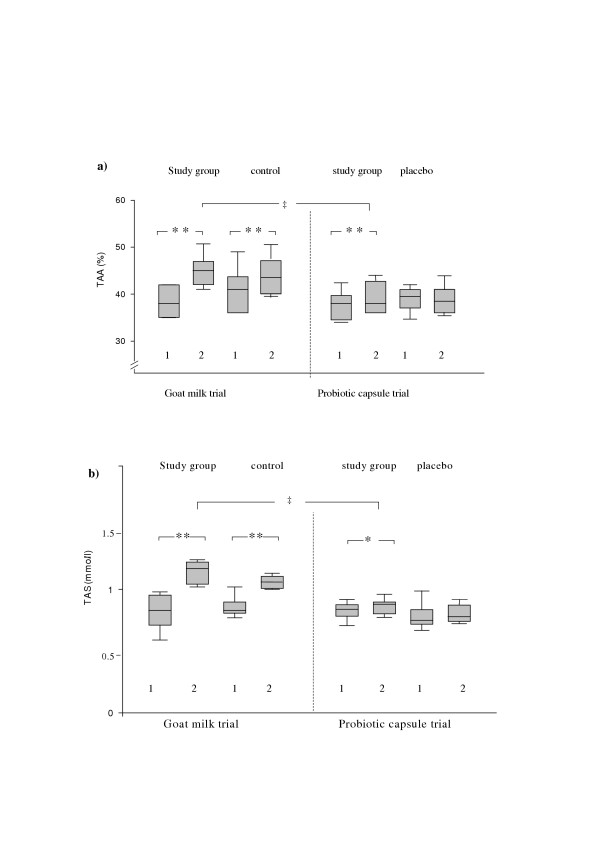
**Effect of ME-3 consumption in fermented goat milk and capsules on human blood oxidative stress markers a) TAA (%) and b) TAS(mmol/l). **1 – Day 0, 2 – Day 21 Significantly different from pre-treatment values: *p < 0.05 (paired t-test); **p ≤ 0.01 (Student's t-test and paired t-test);ME-3 goat milk effect different from the effect of the ME-3 in capsule-form (ANOVA): ‡p ≤ 0.001

The positive effect on the blood ox stress markers as TAA and TAS was seen in the case of both formulations (Fig. 3). Particularly, the additive increase in goat milk group was 6% and 9% respectively as compared to control group, however only 4% for TAA and 2.5% for TAS in probiotic capsule study group as compared to placebo.

The effect of goat-milk consumption on the TAA and TAS values was significantlyhigher (p < 0.001) than by the consumption of the capsulated probiotic (Fig. 3). The decrease of the glutathione red-ox ratio was significant in both groups: the study Group (from 0.15 ± 0.01 to 0.11 ± 0.04 μg/ml, p < 0.01) and control (from 0.14 ± 0.03 to 0.11 ± 0.02 μg/ml, p < 0.01) in the goat milk trial. When the probiotic was consumed in capsulated form, no significant decrease was noticed in the glutathione red-ox ratio.

## Discussion

Our aim was to evaluate the functional efficacy of the antimicrobial and antioxidative probiotic *L. fermentum *ME-3 in normal population with variable food intake. First of all the safety of the *L. fermentum *strain ME-3 was confirmed as no adverse side effects were registered in volunteers. Even relatively high (>10^11 ^CFU) doses of consumed ME-3 had no negative impact on the hosts' general well-being. *Lactobacillus fermentum *as species, used in various food applications, has a well-established history of safe use and is evaluated as GRAS according to the Food and Drug Administration of the USA [21].

Second, a clear improvement of laboratory indices of antioxidative defence system of a healthy host was documented, using both formulations as fermented by *L. fermentum *ME-3 goat-milk and probiotic capsules. This effect was simultaneous with the increase of intestinal lactoflora of healthy volunteers even without necessity for faecal recovery of the strain. In the human population, persons without clinical symptoms have still a quite different health status, including stability, capacity and potency of antioxidative defence to counteract sufficiently to oxidative stress-caused adverse effects [7]. If a probiotic is able to exhibit a positive functionality on oxidative stress-related indices, it helps both to stabilize and promote the potency of the whole body antioxidative defence system in subclinical situations without disease symptoms. That in turn may have an impact for lowering the risk of atherosclerotic damage of blood vessels associated with several cardiovascular and neurodegenerative diseases [22-24].

In our study of healthy volunteers for validation of the antioxidative functionality of probiotic, four well-known oxidative stress markers of blood were chosen [25-27].

The state of the lipid fraction (including also LDL) in the antioxidative defence system of the blood is evaluated by TAA. TAS on the other hand reflects more the antioxidativity of the water-soluble fraction of the human blood. Among the measured blood sera markers both the TAA and TAS values were also improved in the two different study groups. However, there was found a significantly lower improvement of TAA and TAS values in cases of capsule than fermented goat-milk where the recovery of the strains was assessed by AP-PCR.

Similarly, the reduction of the glutathione red-ox ratio was detected after the consumption of fermented by ME-3 goat-milk but not with the capsule. The crucial non-enzymatic cellular antioxidant is GSH [28] present in the millimolar range mainly in the red blood cells, liver, pancreas, kidneys, spleen, eyes, lungs and intestinal cells [29]. The oxidized form of glutathione becomes even at low concentrations toxic, and therefore in the cells the glutathione red-ox ratio is kept as low as possible. In the case of inflammation this balance is shifted towards the oxidized form, indicating non-physiological intracellular oxidative stress.

Thus, our study shows that there is a good association between the mode of formulation of probiotic and expression of its functional properties inside the healthy host. The antioxidative potential of the food supplement containing ME-3 was excellent, as reisolates of the strain from capsule expressed significantly higher TAA in comparison with the base values of the strain in vitro (data not shown). Unexpectedly, the shifts in the antioxidativity markers in blood serum of participants of the probiotic capsule trial were less pronounced in comparison with ME-3 fermented goat milk.

Particularly, the explanation for more expressed positive shifts in oxidative stress markers of volunteers of the fermented goat milk trial could be due to the synergistic effect of the probiotic and the substrate. Milk is not just a carrier for the probiotic *Lactobacillus *strain, but contains natural "lactogenic" factors like lactose, minerals, vitamins and other components that enhance the metabolic activity of ingested probiotic strain in GIT. Both fermented goat milk and goat milk elevated the values of TAA and TAS The goat milk contains different biomolecules (e.g. casomorphins, lactorphins, casokinins, *etc*) having certain antioxidative properties, which can contribute to consumers' plasma antioxidative capacity [30-36]. This was proved by some antioxidative effect also in persons consuming non-fermented goat milk. However, the elevation these indices were remarkably more expressed in the fermented goat milk group, thus the goat milk fermentation with *L. fermentum *ME-3 results in additive increase of total antioxidativity. Therefore, the provisional FAO regulations [37] suggesting the need for health claims by specified formulations of probiotic seem to be of the utmost importance.

Additionally, in our study with experimental fermented milk the average daily dose of *L. fermentum *ME-3 being 11.5 logs CFU was clearly higher than that of capsule (max 9.5 log CFU). It is possible that the dose excesses the amount of bacteria necessary for interacting with intestinal mucosa and the unattached lactobacilli are excreted with faeces. The finding of Saxelin and colleagues confirmed that the faecal recovery of the probiotic strain started from the consumption of more than 9.0 log CFU daily doses of capsulated LGG [38]. To our surprise, in the present study the similar dose did not result in faecal recovery of the strain.

It is possible that the ME-3 strain germinated mainly in some upper parts of intestinal tract where the advantageous conditions for survival and metabolic activity of probiotic lactobacilli were present. Using molecular tools, Marteau *et al*. showed that lactobacilli figuring only 7% of faecal microflora performed up to 30% of microbial communities in human colon [39]. If administered in lower quantities as in case of capsule trial, ME-3 did not reach the detectable level in faecal samples. Yet, its presence in gut was proved by the positive antioxidative health effect in blood but not in urine. Therefore it is understandable that the higher load of metabolically active probiotic bacteria in goat-milk resulted also in their faecal recovery and the highest impact on the oxidative stress indices.

Moreover, in our study the positive impact of ME-3 consumption on the host lactoflora was proved by the increase of faecal lactobacilli counts in all participants of human volunteer studies. In experimental settings the high counts of intestinal lactobacilli have been shown as an important defensive factor against enteric infections [40,41]. Though up to now the period of consumption of probiotics has not been defined, the 3-week ingestion of fermented goat-milk and capsule seemed enough for reaching the aims.

It is important to mention that after consumption of ME-3, a strain with high antagonistic activity, neither the species nor the strain predominated among total lactoflora. This shows a well-granted microbial balance inside the gut, which cannot be disturbed by high load of probiotic bacteria. Apparently, the interconnected advanced metabolism of large gut microbiota keeps the proportions of different species quite stable. Some other investigators have obtained similar results showing the proportional increase of different microbial populations (bifidobacteria, coliforms) after administration of *Lactobacillus *sp. probiotic [42,43].

Thus, the functional efficacy of different formulations of anti-infectious and antioxidative probiotic *L. fermentum *ME-3 were proved both by the increase of the lactobacilli counts providing putative defence against infectious agents in gut and by reduction of the oxidative stress indices of blood and urine of healthy volunteers. Further, studies evaluating the efficacy of ME-3 as adjunct to conventional therapy in patients with atherosclerotic damages and a high-grade oxidative stress are ongoing.

## Conclusion

In non-diseased host, the probiotic health claims can be assessed by improvement of some measurable laboratory indices of well-established physiological functions of organism. In our case, the possibility for augmentation of the antioxidative defence system by the probiotic *L. fermentum *ME-3 in normal population can be proposed.

## Competing interests

Marika Mikelsaar, Mihkel Zilmer, Tiiu Kullisaar, Heidi Annuk (Hynes) and Epp Songisepp are sharing the Estonian patent application: no. EE 2001 00356 29.06.01 and International Patent application: no. WO03002131.

## Authors' contributions

Epp Songisepp, and Pirje Hütt have been in charge of the microbiological analysis. The former has been also in charge of analyzing the results and writing of the manuscript. Jaak Kals was responsible for performance and management of the volunteer trials. Tiiu Kullisaar has been in charge of the biochemical analysis and writing the manuscript. Mihkel Zilmer has conducted the biochemical estimations and writing the manuscript. Reet Mändar has been in charge of the molecular analysis and revising the manuscript. Marika Mikelsaar is the main conductor of the *L. fermentum *ME-3 research; for this paper she has been in charge of the clinical trial design and writing the manuscript.
